# Antimicrobial Stewardship in German non-university hospitals: baseline status and impact of a multifaceted AMS intervention within the prospective ID ROLL OUT study

**DOI:** 10.1007/s15010-025-02658-x

**Published:** 2025-10-06

**Authors:** R. Giesen, G. Först, G. Fink, R. Allen, N. Wimmesberger, D. Hornuss, S. Horn, F. Khaleqi, S. Mertins, M. Schmid, A. Schmidt, T. Tremmel, C. van Uden, F. Wagner, U. Witten-Stephan, Y. Wuwer, P. Mathé, W. V. Kern, E. Farin-Glattacker, S. Rieg

**Affiliations:** 1 Division of Infectious Diseases, Department of Medicine II, Medical Center, Faculty of Medicine, University of Freiburg, Freiburg, Germany; 2 Clinical Pharmacy, Institute of Pharmaceutical Sciences, University of Freiburg, Freiburg, Germany; 3 Section of Health Care Research and Rehabilitation Research, Medical Center, Faculty of Medicine, University of Freiburg, Freiburg, Germany; 4 Department of Medicine 2, Marienhospital Stuttgart, Stuttgart, Germany; 5 Department of Medicine IV, Böblingen Hospital, Clinic Group Southwest, Böblingen, Germany; 6 Department of Medicine II, Schwarzwald-Baar Hospital, Villingen-Schwenningen, Germany; 7 Department of Medicine I, Hegau-Bodensee Hospital Singen, Health Association Landkreis Konstanz, Singen, Germany; 8 Department of Medicine, Hospital Am Eichert (Göppingen), Alb-Fils-Hospitals, Göppingen, Germany; 9 Department of Medicine, Staufer Hospital Schwäbisch Gmünd, Ostalb Hospitals, Mutlangen, Germany; 10 Department of Medicine I, Ostalb Hospital Aalen, Ostalb Hospitals, Aalen, Germany; 11 Department of Medicine I, Sindelfingen Hospital, Clinic Group Southwest, Sindelfingen, Germany; 12 Department of Anesthesia and Intensive Care, Hospital Mittelbaden, Rastatt, Germany; 13 Department of Pneumology, Hospital St. Josef Hospital, Freiburg, Germany

**Keywords:** Antibiotic stewardship (ABS), Antimicrobial stewardship (AMS), Non-university hospital, AMS-GER, Score, Assessment, Sustainability

## Abstract

**Purpose:**

Non-university hospitals are the major provider of inpatient care in Germany, but the extent of antimicrobial stewardship (AMS) activities in this sector is not well known. We aimed to evaluate the implementation of AMS in ten non-university hospitals in Germany.

**Methods:**

A pre-existing French score covering key AMS categories (structures, resources and actions) was adapted to the German AMS guidelines and named AMS-GER score. The score was assessed before, during and after the implementation of a bundle of AMS measures. The bundle was implemented as part of the ID ROLL OUT study – a multicentre pre-post interventional study in non-university hospitals in Germany.

**Results:**

At baseline, the median AMS-GER score was 37% (range 20–60%), indicating poor implementation in 9 out of 10 hospitals. The intervention resulted in a significant score improvement to 76% (range: 62–86%, p < 0.001). At the one-year follow-up after the intervention, the AMS-GER score had decreased in all hospitals (median: -13 percentage points (range: 48–80%, p = 0.015)). Hospitals with ongoing full-time AMS/ID staffing (4/10 hospitals) experienced a smaller decrease ( – 13 percentage points) than those without ( – 32 percentage points in 6/10 hospitals).

**Conclusion:**

Routine integration of AMS in a large sample of non-university hospitals in Germany is low but can be significantly improved by targeted interventions. The decline in the AMS-GER score in the follow-up phase—particularly in hospitals lacking ongoing AMS/ID staffing—highlights the need for sustained staffing and systematic benchmarking. In this context, the AMS-GER score offers a structured tool for AMS monitoring in German hospitals.

**Supplementary Information:**

The online version contains supplementary material available at 10.1007/s15010-025-02658-x.

## Introduction

The global burden of disease due to bacterial resistance to antimicrobial agents is considerable and poses a challenge to public health [1]. Non-reflected use of antimicrobials (AM) contributes to the increasing development of resistance. Antimicrobial stewardship (AMS) programmes intend to ensure the rational use of AM. These programmes can reduce the overall consumption of AM, thereby aiming to slow the development of antimicrobial resistance and improving patient care. [2–4]. In contrast to other European countries there is no systematic assessment of the extent of AMS measures in German hospitals. Despite the existence of a comprehensive national guideline [5] and a widespread AMS education initiative, the implementation of AMS programmes in German hospitals is inconsistent and often limited. Particularly in the non-university hospital setting, the degree of AMS implementation seems to be lower [6]. Furthermore AMS studies in non-university hospitals are underrepresented compared to large, academic and tertiary care institutions, although the vast majority of hospital beds in Germany (89% of acute care beds) are in the non-university sector [7].

The objective of this longitudinal survey study was to quantify the extent to which AMS is implemented in ten non-university hospitals in Germany, prior to, during, and after the implementation of a differentiated AMS intervention study (ID ROLL OUT study) [8].

## Methods

### Study design and setting

This longitudinal survey study was conducted parallel to the ID ROLL OUT study– a multicentre, two-armed, interventional study on implementing AMS measures and infectious diseases specialist services (IDS) in ten non-university hospitals in Germany. The participating acute care hospitals varied in size and structure, with bed capacities ranging from 260 to 835, with a median of 401 beds (Table 1). The participating hospitals represented approximately 10% of all hospital beds in the federal state of *Baden-Württemberg*, which has a population of 11.1 million. The hospitals were geographically distributed over the federal state, including rural and urban settings.

**Table 1:** Hospital characteristics

	Number of beds	Number of departments	Sponsorship
Hospital 1	260	4	Public
Hospital 2	282	9	Private
Hospital 3	335	9	Public
Hospital 4	375	9	Public
Hospital 5	400	10	Public
Hospital 6	401	13	Public
Hospital 7	465	10	Public
Hospital 8	639	16	Public
Hospital 9	761	18	Charitable
Hospital 10	835	19	Public
Beds all hospitals (median, range)	401 (260–835)		

### ID ROLL OUT intervention

The AMS-GER score was assessed before, during and after the implementation of AMS/ID interventions carried out as part of the ID ROLL OUT study. The ID ROLL OUT study used two intervention strategies: In study arm 1 (AMS) a multi-faceted AMS intervention was developed and implemented at the structural, organisational and personal levels. In addition, telephone advice by infectious diseases (ID) specialists was available during normal working hours. The intensity of the intervention was stepped up and supplemented by on-site services provided by ID specialists (IDS) for study arm 2 (AMS + IDS). In all participating hospitals, the AMS teams consisted of doctors with specific training in antimicrobial stewardship (study arm 1: AMS) or infectious diseases specialists (study arm 2: AMS + IDS), together with hospital pharmacists, who underwent specific training in AMS as well. The intervention was carried out by existing hospital staff who were trained within the framework of the study and specifically released from their regular duties to participate in the intervention. In the follow-up phase one year after the intervention phase study-related support was discontinued, and hospitals had no further external financial or personal support. Only 4/10 hospitals had independently allocated permanent AMS/ID staffing in the follow-up phase.

**Fig. 1:**
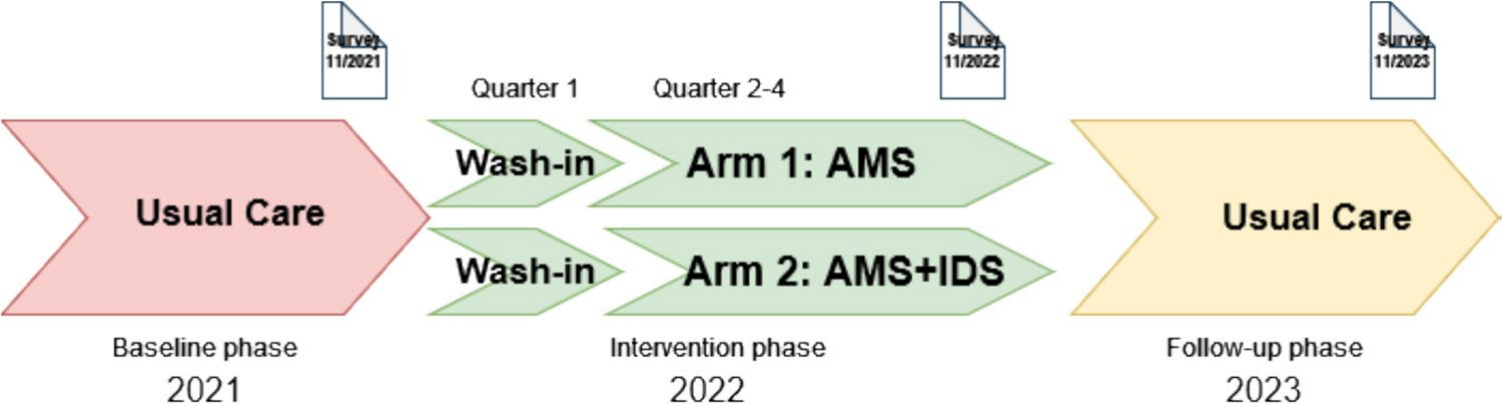
Visualization of the survey time points within the ID ROLL OUT study

Specific infection types treated within in study setting can be extrapolated from point prevalence analyses, which were carried out three times in the baseline phase and three times in the intervention phase as part of the ID ROLL OUT study. The most commonly treated infections were respiratory (17%), abdominal (15%), and urinary tract and skin and soft tissue infections (12% each). 20% of the prescribed antibiotics were used for surgical or medical prophylaxis [9]. Hospital characteristics, such as the number of beds and types of departments determined the allocation to each study arm. After a preparatory baseline phase in 2021 the interventions were implemented in 2022 (with a wash-in phase in quarter 1). A summary of the ID ROLL OUT study interventions is shown in Supplementary Table 1 and the study procedure is graphically depicted in Fig. 1. The ID ROLL OUT study protocol has been published previously [8].

### Score development and data collection

The ICATB.2 score (composite indicator for proper use of antibiotics—2nd Version) is a French assessment tool which measures the degree of practical implementation of AMS at hospital level [10]. It comprises 27 questions and queries key AMS-categories (structures/framework, resources, and actions) (see Supplementary Table 2a and 2b). In order to quantify the practical implementation of AMS as accurately as possible in our study setting, we adapted the ICATB.2 score to the German setting and recommendations (e.g. German AMS guideline) and named it the AMS-GER score. The structure and weighting were preserved from the ICATB.2 score, which had been applied nationwide in France until 2018, with annual assessment of all hospitals and public reporting by the French health authorities. The total score ranges from 0 to 100 points and is expressed in percent. Each hospital is assigned an AMS performance level (A to E—very good to very poor) according to the score and depending on the size of the hospital (Table 2). There are five subcategories within the score: The "framework" sub-category (four questions, max. 16 points) describes the structural requirements for AMS activities. The next subcategory covers AMS resources, which include both infrastructure and human resources (7 questions, 38 points). The remaining subcategories address AMS actions that represent preventative (5 questions, 18 points), surveillance (5 questions, 10 points) and evaluation (6 questions, 18 points) tools.

**Table 2:**
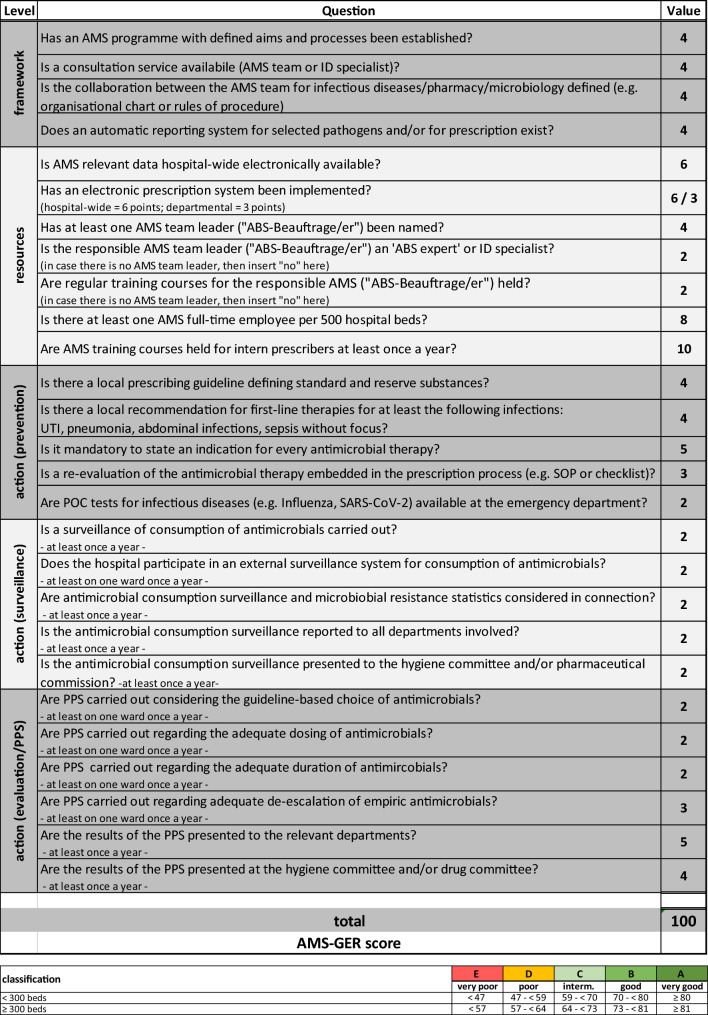
AMS-GER score

*ABS* Antibiotic stewardship, *AMS* Antimicrobial stewardship, *ID* infectious diseases, *POC* point of care, *PPS* point prevalence survey, *SOP* Standard operating procedure, *UTI* urinary tract infection

The information required to determine the AMS-GER score was completed by study team members from each of the ID ROLL OUT hospital sites. To evaluate the status quo and the lasting impact of the interventions, the survey was conducted at three time points: before (11/2021), at the end of (11/2022), and one year after the intervention phase (11/2023, see Fig. 1).

### Subcategory analyses

In addition to the evaluation of the overall score, the five subcategories were analysed for each of the three time points: baseline, intervention and follow-up phase. As guidelines emphasise the importance of adequate AMS staffing for sustainability, a comparative analysis was carried out. It compared hospitals which employed at least one AMS staff member on a full-time equivalent (FTE) basis per 500 hospital beds during the follow-up phase, with those that did not. In order to ensure comparability, the question on FTE was excluded for this partial analysis. As point prevalence surveys (PPS) were an integral part of the AMS interventions, a separate AMS-GER score was calculated excluding the PPS questions as a sensitivity analysis. In addition, comparative analyses were performed with regard to hospital size and the ID ROLL OUT intervention strategies (AMS vs. AMS + IDS). Small hospitals were defined as < 350 beds (n = 3), medium-sized hospitals as 350–600 beds (n = 4) and large hospitals as > 600 beds (n = 3).

### Statistical analysis

Non-normally distributed data were displayed as median and interquartile range (IQR). Categorical variables were reported as proportions. For the comparison of the three time points of the AMS-GER score a paired Wilcoxon test was used. For categorical comparison between different hospital sizes, the Mann–Whitney U Test was used. P-values below 0.05 were considered to be statistically significant. Statistical analysis was performed using R Studio (R version 4.2.2).

## Results

### AMS-GER score: Baseline vs. intervention phase

**Fig. 2:**
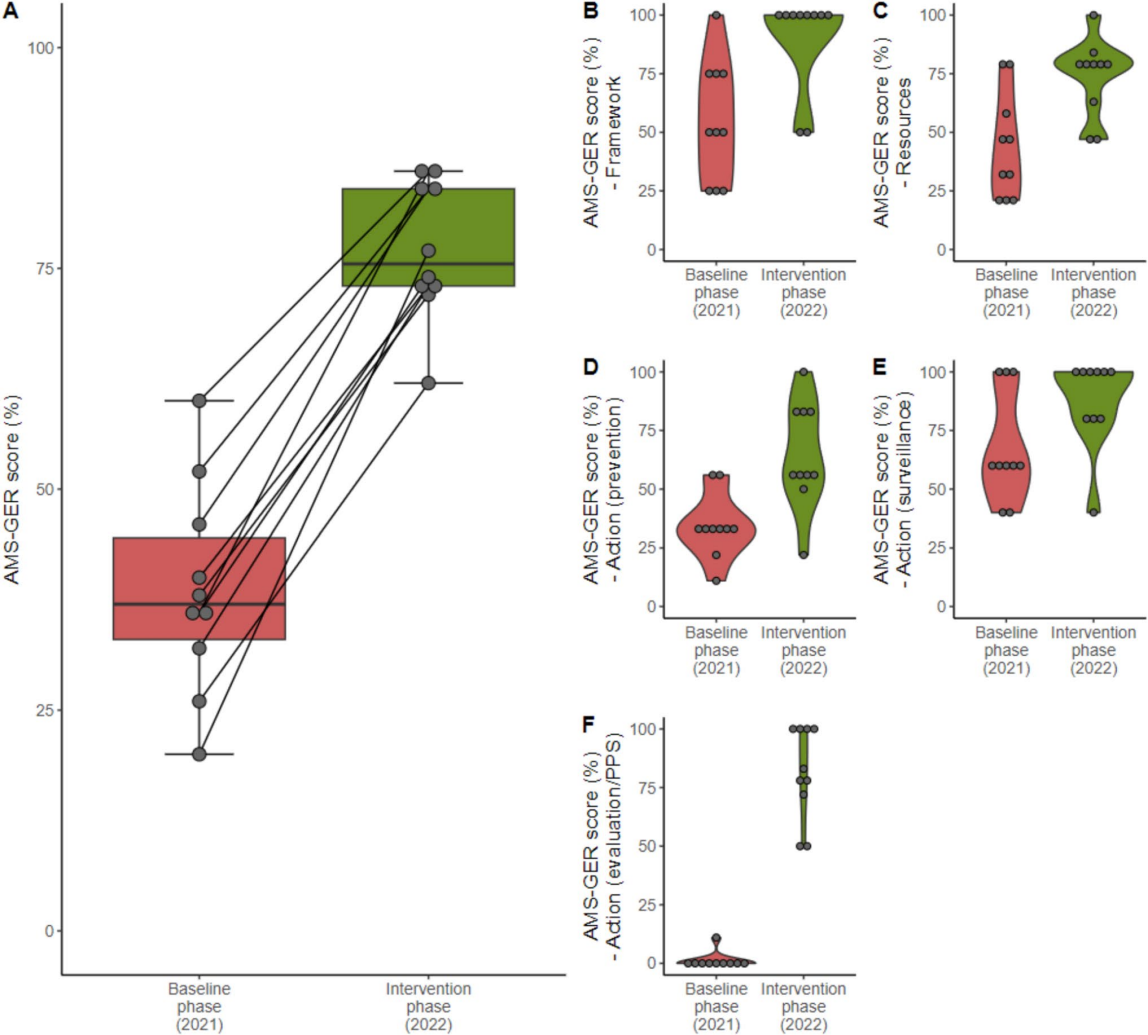
AMS-GER score baseline vs. intervention phase. **A** Total AMS-GER score baseline vs. intervention phase, **B** Subcategory: framework, **C** Subcategory: resources, **D** Subcategory: action (prevention), **E** Subcategory: action (surveillance) **F** Subcategory: action (evaluation)

Each hospital completed the survey for the baseline phase (2021), intervention phase (2022) and follow-up phase (2023). At the end of the baseline phase, the median value of the AMS-GER score of the participating hospitals was 37% (range: 20–60%). One hospital achieved performance level D, nine out of ten hospitals were assigned the lowest performance-level E. At the end of the intervention phase, the AMS-GER score and corresponding performance level improved in all the hospitals. Four hospitals now had the highest performance level A. Five hospitals achieved level B and one hospital level D. The median AMS-GER score improved significantly to 76% (range: 62–86%) with a median increase of + 39 percentage points (range: 26–57%, p < 0.001), corresponding to an overall performance-level B. An improvement was observed in all five subcategories. In the “framework” subcategory eight out of ten hospitals improved to 100%. (Fig. 2). The most prominent improvement was seen in the "actions—evaluation" category which included PPS activities. By excluding the questions related to PPS activities, the total AMS-GER score still improved to 76% (range: 65–84%), with the median increase remaining significant at + 31 percentage points (range: 10–48%, p = 0.006) (Supplementary Fig. 1). No significant difference was found as regards both intervention strategies (AMS vs. AMS + IDS) (Supplementary Fig. 2).

### AMS-GER score: Intervention vs. follow-up phase

**Fig. 3:**
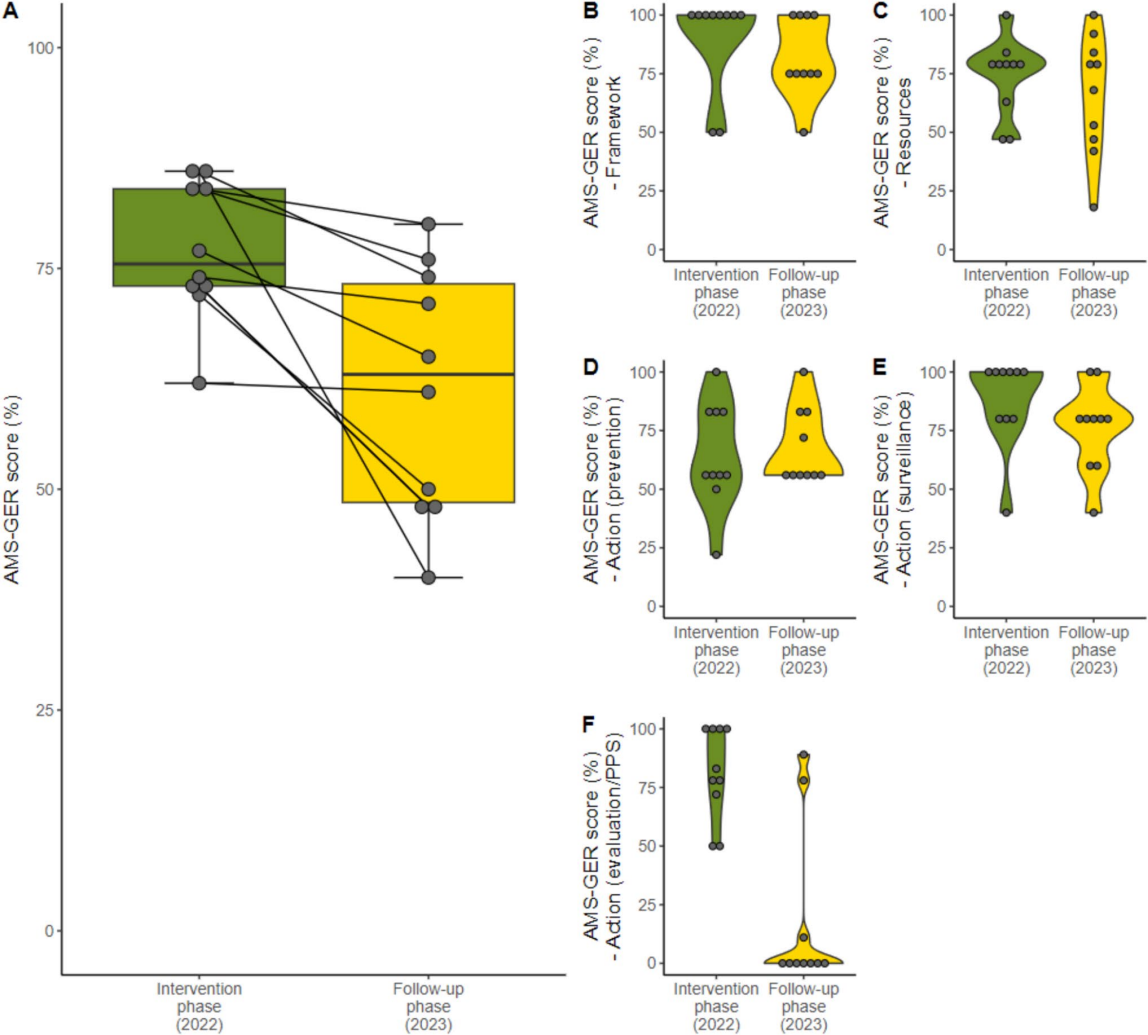
AMS-GER score intervention vs. follow-up phase. **A** Total AMS-GER score intervention vs. follow-up phase, **B** Subcategory: framework, **C** Subcategory: resources, **D** Subcategory: action (prevention), **E** Subcategory: action (surveillance) **F** Subcategory: action (evaluation)

In the follow-up phase one year after the intervention, the AMS-GER score dropped in all the hospitals. No hospital reached the highest performance-level A. Three hospitals performed at level B, two at level C, three at level D and two hospitals achieved the lowest level E. One hospital maintained its performance-level. Compared to the intervention phase the median AMS-GER score dropped to 63% (range: 48–80%) with a median decrease of  – 13 percentage points (range: 1–46%, p = 0.015), which corresponds to an overall performance level C (for hospitals < 300 beds) and D (for hospitals > 300 beds). None of the subcategories improved in the follow-up phase. One of the five subcategories ("actions (prevention)”—D) remained stable. All the other subcategories decreased, most prominently the “action – evaluation” subcategory (Fig. 3). No significant difference was found, as regard the two study arms, between intervention and follow-up phase (Supplementary Fig. 2).

### AMS-GER score: Baseline vs. follow-up phase and effect of ongoing AMS/infectious diseases staffing and hospital size

In the comparison between the baseline phase (2021) and the follow-up phase (2023), the AMS-GER score remained significantly higher than at baseline. The median score increased from 37% (range: 20–60%) at baseline phase to 63% (range: 48–80%) at follow-up phase, corresponding to a median difference of + 26 percentage points (p = 0.006, Supplementary Fig. 3). While all hospitals showed improvement compared to the baseline phase, the performance levels varied: three hospitals achieved level B, two level C, three level D, and two remained at level E. Given this improvement compared to the baseline phase, we next analysed the impact of ongoing AMS/ID staffing levels.

At the baseline phase, none of the hospitals had specialised AMS/ID staffing. Four Hospitals continued to have an FTE basis per 500 hospital beds specifically dedicated to AMS/ID in the follow-up phase. These hospitals with ongoing staffing had a median AMS-GER score (FTE question excluded) of 34% (range: 22–65%) at baseline, 78% at intervention (range: 67–85%) and 65% (range: 58–72%) at follow-up phase. This corresponds to a median difference of + 44 percentage points (range: 20–53%) between the baseline and intervention phase, -13 percentage points (range: 9–13%) between the intervention and follow-up phase, and + 31 percentage points (range: 7–40%) between the baseline and follow-up phase.

**Fig. 4:**
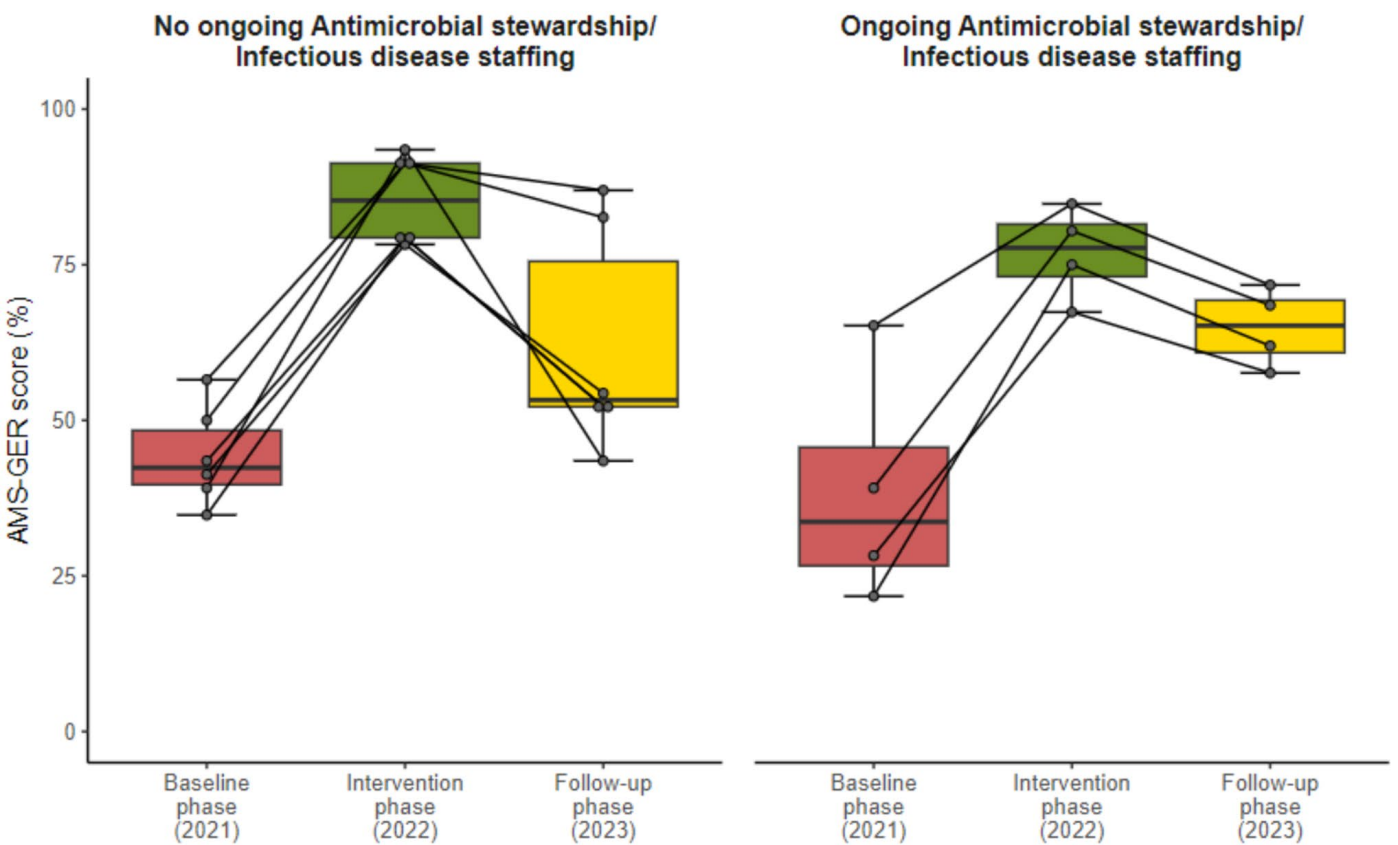
AMS-GER score (question on FTE excluded) for ongoing AMS/ID staffing vs. no ongoing AMS/ID staffing; baseline vs. follow-up phase

The six hospitals without ongoing staffing had a median AMS-GER score of 42% (range: 35–57%) at baseline, 85% during the intervention (range: 78–93%), and 53% (range: 43–87%) at the follow-up phase. This corresponds to a median difference of + 43 percentage points (range: 34–54%) between the baseline and intervention phase, -32 percentage points (range: 4–50%) between the intervention and follow-up phase, and + 11 percentage points (range: 4–33%) between the baseline and follow-up phase. In the comparison between baseline and follow-up phases, hospitals with ongoing AMS/ID staffing showed a 20 percentage point higher AMS-GER score than hospitals without permanent staffing (Fig. 4). When stratified by hospital size (< 350 beds, 350–600 beds, > 600 beds), larger hospitals showed higher AMS-GER scores at the baseline phase. From baseline to follow-up phase, small hospitals demonstrated a median increase of + 10 percentage points (range: 4–12%), compared to + 34 percentage points (range: 16–35%) in medium-sized hospitals and + 24 percentage points (range: 14–30%) in large hospitals (Supplementary Fig. 4).

## Discussion

The current study evaluated the extent of AMS implementation in non-university hospitals – representing the majority of acute care facilities in Germany—in the context of a multifaceted AMS/ID intervention across three time points: baseline, intervention, and one-year follow-up by using an established score adapted to the German health care context (AMS-GER score).

Our main findings are as follows:

1. According to the AMS-GER score a very low level of AMS implementation was observed in the baseline phase, with nine out of ten hospitals performing at the lowest level (E).
2. The intervention strategies led to a significant improvement in the AMS-GER score, which increased by more than twofold compared to the baseline.
3. In the one-year follow-up after the intervention we observed a significant decrease in the AMS-GER score. However, hospitals which maintained adequate FTE AMS/ID staffing showed a smaller score reduction despite starting from a lower baseline.

The low level of AMS implementation in the baseline phase indicates that the framework, resources and actions for AMS are still put into action to a limited extent in routine care in non-university hospitals in Germany. Our observations are consistent with the results of a 2017 survey of 164 acute hospitals in Germany, which found that non-university hospitals had less well-developed AMS structures than university hospitals [6]. In other European countries, implementation appears to be significantly higher than in our study setting, as was shown for acute hospitals in Italy, France, Slovenia and the Netherlands in 2017 [11]. Yet, these countries showed relevant differences in terms of the presence of AMS prerequisites, targets and improvement strategies, reflecting the heterogeneity of AMS implementation even within Europe. Although the ID ROLL OUT intervention strategies resulted in improvement at all AMS levels, a short-term one year follow-up outside of the study setting revealed a significant decline in the AMS-GER score, emphasising the need for regular, systematic, ongoing assessment to not only enable benchmarking, but also identify areas for improvement. Several countries have recognised the necessity of systematic AMS assessment and have established national evaluation frameworks. In the United States, for example, a mandatory annual survey for all hospitals includes a relevant section on AMS implementation, which provides a structured mechanism for evaluating and promoting stewardship efforts. The impact of such initiatives is evidenced by the fact that the proportion of acute care hospitals meeting the CDC core elements of hospital antibiotic stewardship programmes has increased significantly from 41% in 2014 to 95% in 2021 [12]. Similarly in an obligatory French nationwide survey from 2017 (2.059 hospitals) the median ICATB.2 score, which forms the basis of the AMS-GER score, was 84%, indicating a widespread AMS implementation [10].

There are international efforts towards a single standardised assessment tool for hospital AMS programmes [13] However, national evaluation systems are needed to complement this, reflecting national requirements and recommendations. For example the ongoing Belgian BEAST initiative (Belgian Evaluation of Antimicrobial Stewardship Teams) aims to assess the current state of AMS, both at national and local levels, with the goal of offering recommendations to enhance AMS efficiency [14]. To date, there is no uniform, standardised recording of AMS in German hospitals. The AMS-GER score, developed and applied in this study, could provide a structured and feasible approach to evaluate AMS efforts and could serve as a foundation for a national monitoring framework in Germany. Without regular evaluation and benchmarking, there is a risk that AMS efforts remain inconsistent, and improvements observed through interventions – such as those in our study – are not maintained over time. A legally binding national benchmarking system would ensure that hospitals are continuously monitored and held accountable for maintaining AMS structures and processes. Such an approach could be incorporated into existing hospital accreditation frameworks or linked to quality-based incentives – strategies that have proven effective in other contexts, as demonstrated by a recent Japanese study, in which sustainable improvements in outpatient antibiotic consumption were achieved through financial incentives [15].

Implementing an effective and sustainable AMS programme requires considerable time and effort, making adequate staffing and funding essential prerequisites. Countries such as the Netherlands, where a significantly higher proportion of personnel is dedicated to AMS and supportive resources and organisational structures are available in nearly every hospital, demonstrate a substantially greater likelihood of implementing AMS measures [16]. This association is also supported by a study in the US showing that financial support independently predicts a hospital’s adherence to all seven CDC core elements of AMS programmes [17]. Our observations from the follow-up phase after the ID ROLL OUT study underscore the challenges of maintaining AMS without continuous resources. Outside the controlled study setting the staffing recommendation of 1 FTE/500 hospital beds required by the German AMS guidelines [5] was met by only four of ten hospitals. The other hospitals did not continue the staffing due to limited resources and no legal obligation to ensure this standard. Notably, the hospitals, with a continuous full-time AMS/ID FTE, showed a lesser decline (− 13 percentage points) in the AMS-GER score at follow-up than hospitals without ongoing staffing (-32 percentage points), despite starting from a lower baseline. In hospitals without sustained AMS/ID FTEs, stewardship became an additional duty for existing staff rather than a protected responsibility, which likely reduced available time and contributed to the decline in AMS-GER scores. When stratifying by hospital size, larger institutions tended to start with higher AMS-GER scores and showed more sustained implementation at follow-up compared to smaller hospitals. In line with this larger hospitals consistently reported greater uptake of the CDC’s Core Elements and implementation of key stewardship measures compared to smaller hospitals, although the gap has narrowed over time [12]. However, as all hospitals with ongoing AMS/ID staffing were medium-sized or large, the observed effect of hospital size is likely confounded by the availability of dedicated AMS resources. These findings highlight the critical role of sustained human and financial resources in ensuring the long-term implementation of AMS. In addition to scientific evaluations and clinical studies in AMS, which are of major importance, the implementation of AMS measures in routine care is pivotal but not easy to accomplish, as even in hospitals that served as ID ROLL OUT study sites, there was a significant decrease over time. Our findings therefore also highlight the need for further research to determine the long-term effects and sustainability of AMS interventions. The effectiveness of AMS programmes in reducing the AM use and related costs is well documented [2]. Previous studies in other settings have demonstrated a correlation between AMS scores and AM use [18, 19]. Based on these findings, we hypothesize that higher AMS-GER scores are associated with more appropriate AM use. Future studies should test this hypothesis – analyses within the ID ROLL OUT study framework are under way. Notably, time-consuming activities such as point prevalence analyses were rarely carried out in the follow-up after the end of the ID ROLL OUT study. This highlights the potential for automated approaches which extract and analyse data from electronic health records and may therefore be a promising tool in the future to support AMS teams [20]. Prospectively, procedures such as AM monitoring or AM prescription analyses could be supported by AI-related processes, which would particularly support hospitals with limited resources. Yet, these tools may prove to be efficient in the context of surveillance, however, other AMS measures like education, audit and feedback, AMS rounds etc. will still depend on personal interaction making adequate staffing levels essential even with further progress in automated software tools. Both study arms (AMS and AMS + IDS) showed significant improvement during the intervention, with no significant difference between them. This should be interpreted with caution, as the AMS-GER score reflects structural requirements rather than clinical outcomes. Potential additional effects of IDS input are therefore not reflected in the AMS-GER score.

The present study has several strengths. Firstly, the developed AMS-GER score provides a standardised framework to assess and benchmark AMS activities in German hospitals. Although the AMS-GER score has not been validated so far, the score is based on a preexisting instrument which has already been successfully used on a large scale in France. Moreover, the multicentric evaluation of a large subset of non-university hospitals in Germany with different size, structure, and sponsors/owners increases the likelihood of the generalisability of our results. Another strength is the longitudinal design which allows evaluation of both short- and medium-term sustainability of the implemented AMS measures.

However, limitations inherent to the study design have to be taken into consideration. The AMS-GER score provides a structured approach to evaluating AMS activities, yet a single scoring system has limitations – differentiated qualitative aspects of AMS implementation such as quality AM-prescribing decisions or impact on patient outcomes are not presented. Additional outcomes measures as case-mix, length of hospital stay, and economic aspects were assessed within the ID ROLL OUT study framework, these analyses will be reported separately as the present manuscript focuses on structural prerequisites for AMS as measured by the AMS-GER score. Furthermore, additional barriers such as prescribers attitude, which is well described in the literature [21], were not captured by the AMS-GER score and therefore could not be evaluated in our analysis. These aspects will be addressed in a separate qualitative evaluation. Self-reported data from local hospital staff has the potential for bias, as staff may over- or underestimate the adherence to AMS quality measures. The follow-up assessment was performed one year after the intervention. While this period provides valuable insights into medium-term effects, it may still be too short to fully capture the long-term sustainability of AMS measures. Extended follow-up data are not yet available; however, the ID ROLL OUT framework offers the opportunity for future evaluations over several years. The AMS-GER score was used in non-university hospitals without established AMS programmes in this study. Future applications on a larger scale (e.g. nationwide) especially in hospitals with established AMS structures, such as university hospitals, would be desirable. The ID ROLL OUT study could not randomise participating hospitals to the two arms (AMS vs. AMS + IDS), instead centres were stratified according to size and (sub)specialities, therefore potential selection bias cannot be excluded. However, a comprehensive adjustment for hospital characteristics was carried out and the hospitals in the two study arms had a similar AMS-GER score at baseline. Furthermore, the interventions took place during the Covid-19 pandemic, with an unclear exerted effect on AMS measures.

## Conclusion

Our findings demonstrate that routine integration of AMS in a large sample of non-university hospitals in Germany is low but can be significantly improved by targeted interventions. However, the decline in the AMS-GER score observed in the follow-up phase outside the study setting highlights the need for sustained support. To counteract the decline of AMS measures observed in some hospitals solutions are required at both the institutional and clinical level. At the institutional level anchored stewardship positions, secure financing for dedicated AMS/ID staffing beyond projects and regular benchmarking through tools are needed. In this context, the AMS-GER score offers a structured tool for AMS monitoring in German hospitals. At the clinical level, continuous education, audit-and-feedback mechanisms, and digital support tools (including emerging AI-based solutions) are essential to maintain engagement. Future research should focus on the long-term effectiveness and on the validation of the proposed AMS-GER score and its correlation with AM use, prescription quality and patient outcomes.

## Supplementary Information

Below is the link to the electronic supplementary material.

Supplementary file1 (DOCX 580 KB)

## Data Availability

Anonymized data is available upon request to the corresponding author.

## References

[1] Murray CJL, Ikuta KS, Sharara F, Swetschinski L, Robles Aguilar G, Gray A, et al. Global burden of bacterial antimicrobial resistance in 2019: a systematic analysis. Lancet. 2022;399:629–55. 10.1016/S0140-6736(21)02724-0. 35065702 10.1016/S0140-6736(21)02724-0 PMC8841637

[2] Karanika S, Paudel S, Grigoras C, Kalbasi A, Mylonakis E. Systematic review and meta-analysis of clinical and economic outcomes from the implementation of hospital-based antimicrobial stewardship programs. Antimicrob Agents Chemother. 2016;60:4840–52. 10.1128/AAC.00825-16. 27246783 10.1128/AAC.00825-16 PMC4958232

[3] Schuts EC, Hulscher MEJL, Mouton JW, Verduin CM, Stuart JWTC, Overdiek HWPM, et al. Current evidence on hospital antimicrobial stewardship objectives: a systematic review and meta-analysis. Lancet Infect Dis. 2016;16:847–56. 10.1016/S1473-3099(16)00065-7. 26947617 10.1016/S1473-3099(16)00065-7

[4] Barlam TF, Cosgrove SE, Abbo LM, MacDougall C, Schuetz AN, Septimus EJ, et al. Implementing an antibiotic stewardship program: Guidelines by the infectious diseases Society of America and the Society for Healthcare Epidemiology of America. Clin Infect Dis. 2016;62:e51-77. 10.1093/cid/ciw118. 27080992 10.1093/cid/ciw118 PMC5006285

[5] de With K, Allerberger F, Amann S, Apfalter P, Fellhauer M, Geiss HK, et al. Strategies to enhance rational use of antibiotics in hospital: a guideline by the German Society for Infectious Diseases. Infection. 2016. 10.1007/s15010-016-0885-z. 27066980 10.1007/s15010-016-0885-z PMC4889644

[6] Först G, Fellhauer M, Hug MJ, Probst W, Ranft D. Antibiotic Stewardship in ­deutschen Krankenhäusern. Krankenhauspharmazie. 2018;39:304–10.

[7] Statistischer Bericht Grunddaten der Krankenhäuser 2022. [Statistical report. Basic data of hospitals 2022]. Wiesbaden: Federal Statistical Office of Germany; 2023 [Internet]. Federal Statistical Office of Germany. [cited 2025 Jun 23]. https://www.destatis.de/DE/Themen/Gesellschaft-Umwelt/Gesundheit/Krankenhaeuser/_inhalt.html. Accessed 23 Jun 2025

[8] Zimmermann N, Allen R, Fink G, Först G, Kern WV, Farin-Glattacker E, et al. Antimicrobial stewardship with and without infectious diseases specialist services to improve quality-of-care in secondary and tertiary care hospitals in Germany: study protocol of the ID roll out study. Infect Dis Ther. 2022;11:617–28. 10.1007/s40121-021-00552-1. 34751941 10.1007/s40121-021-00552-1 PMC8576457

[9] Först G, Giesen R, Fink G, Sehlbrede M, Wimmesberger N, Allen R, et al. An in-depth analysis of antimicrobial prescription quality in 10 non-university hospitals, in southwest Germany, 2021. Eurosurveillance [Internet]. 2024 [cited 2025 Jan 9];29. 10.2807/1560-7917.ES.2024.29.46.2400156. 10.2807/1560-7917.ES.2024.29.46.2400156 PMC11565651 39544144

[10] Indicateurs de qualité et de sécurité des soins - Infections associées aux soins - recueil 2018 [Internet]. [cited 2025 Jun 23]. https://www.data.gouv.fr/en/datasets/indicateurs-de-qualite-et-de-securite-des-soins-infections-associees-aux-soins-recueil-2018/. Accessed 23 Jun 2025.

[11] Kallen MC, Binda F, Ten Oever J, Tebano G, Pulcini C, Murri R, et al. Comparison of antimicrobial stewardship programmes in acute-care hospitals in four European countries: a cross-sectional survey. Int J Antimicrob Agents. 2019;54:338–45. 10.1016/j.ijantimicag.2019.06.005. 31200022 10.1016/j.ijantimicag.2019.06.005

[12] O’Leary EN, Neuhauser MM, McLees A, Paek M, Tappe J, Srinivasan A. An update from the National Healthcare Safety Network on hospital antibiotic stewardship programs in the United States, 2014–2021. Open Forum Infect Dis. 2024;11:ofad684. 10.1093/ofid/ofad684. 38344128 10.1093/ofid/ofad684 PMC10854390

[13] Patel T. Global Antibiotic Stewardship Evaluation Tool (G-ASET) for Inpatient Healthcare Facilities. CDC. 2024.

[14] Belgian Evaluation of Antimicrobial Stewardship Teams [Internet]. sciensano.be. [cited 2025 Jun 24]. https://www.sciensano.be/en/projects/belgian-evaluation-antimicrobial-stewardship-teams. Accessed 24 Jun 2025

[15] Okubo Y. Long-Term Effectiveness of Financial Incentives for Not Prescribing Unnecessary Antibiotics to Children With Acute Respiratory and Gastrointestinal Infections: Japan’s Nationwide Quasi-Experimental Study. 10.1093/cid/ciae577 39579079

[16] Kallen MC, Ten Oever J, Prins JM, Kullberg BJ, Schouten JA, Hulscher MEJL. A survey on antimicrobial stewardship prerequisites, objectives and improvement strategies: systematic development and nationwide assessment in Dutch acute care hospitals. J Antimicrob Chemother. 2018;73:3496–504. 10.1093/jac/dky367. 30252063 10.1093/jac/dky367

[17] O’Leary EN, Van Santen KL, Webb AK, Pollock DA, Edwards JR, Srinivasan A. Uptake of antibiotic stewardship programs in US acute care hospitals: findings from the 2015 national healthcare safety network annual hospital survey. Clin Infect Dis. 2017;65:1748–50. 10.1093/cid/cix651. 29020178 10.1093/cid/cix651

[18] Langford BJ, Wu JH-C, Brown KA, Wang X, Leung V, Tan C, et al. Assessing the impact of antibiotic stewardship program elements on antibiotic use across acute-care hospitals: an observational study. Infect Control Hosp Epidemiol. 2018;39:941–6. 10.1017/ice.2018.121 10.1017/ice.2018.121 29893654

[19] Pakyz AL, Moczygemba LR, Wang H, Stevens MP, Edmond MB. An evaluation of the association between an antimicrobial stewardship score and antimicrobial usage. J Antimicrob Chemother. 2015;70:1588–91. 10.1093/jac/dku555. 25614043 10.1093/jac/dku555 PMC4481682

[20] Renggli L, Plüss-Suard C, Gasser M, Sonderegger B, Kronenberg A. Assessing the conversion of electronic medical record data into antibiotic stewardship indicators. J Antimicrob Chemother. 2023. 10.1093/jac/dkad235. 37527399 10.1093/jac/dkad235 PMC10477111

[21] Zetts RM, Stoesz A, Garcia AM, Doctor JN, Gerber JS, Linder JA, et al. Primary care physicians’ attitudes and perceptions towards antibiotic resistance and outpatient antibiotic stewardship in the USA: a qualitative study. BMJ Open. 2020;10:e034983. 10.1136/bmjopen-2019-034983. 32665343 10.1136/bmjopen-2019-034983 PMC7365421

