# Supplementary material for: Antimicrobial Stewardship in German non-university hospitals: baseline status and impact of a multifaceted AMS intervention within the prospective ID ROLL OUT study
Source: Infection. 2025 Oct 6;54(1):263–73. doi: 10.1007/s15010-025-02658-x (PMC12864315; doi:10.1007/s15010-025-02658-x)
Supplement: Supplementary file 1 — Supplementary file1 (DOCX 580 KB) [file 15010_2025_2658_MOESM1_ESM.docx]

**Supplementary Table 1** Comparison of ID ROLL OUT study interventions

| Study arm 1  (AMS) | Study arm 2  (AMS+IDS) |
| --- | --- |
| **Personnel resources** for AMS team members [in full-time equivalents (FTE)]:  Medical: 0.4 FTE (hospitals < 500 beds) or 0.6 FTE (hospitals > 500 beds)  Pharmaceutical: 0.3 FTE (hospitals < 500 beds) or 0.4 FTE (hospitals > 500 beds)  **In-house guidelines**  for common infectiological clinical pictures and anti-infectives including dose recommendations  **Restriction** and prescription authorisation for reserve substances; release by AMS team  **Prescription audits**  (point prevalence survey at hospital level every 3 months)  **Training events** for prescribers  (1 x 10 min. lecture per department; 1-2 x 45 min. workshops per hospital)  Provision of **posters and pocket cards** for prescribers  **Ward rounds** (intensive care units, wards with a high density of anti-infective prescriptions) 1-2 x per week  Counselling by IDS via telephone  (Mon. - Fri. 8am – 5pm) | **Personnel resources** for AMS team members [in full-time equivalents (FTE)]:  Medical: 0.8 FTE (hospitals < 500 beds) or 1.0 FTE (hospitals > 500 beds)  Pharmaceutical: 0.4 FTE (hospitals < 500 beds) or 0.5 FTE (hospitals > 500 beds)  **In-house guidelines**  for common infectiological clinical pictures and anti-infectives including dose recommendations  **Restriction** and prescription authorisation for reserve substances, release by ID after consultation  **Prescription audits**  (point prevalence survey at hospital level every 3 months)  **Training events** for prescribers  (3 x 10 min lecture per department; >3-4 x 45 min workshops per hospital)  Provision of **posters and pocket cards** for prescribers  **Ward rounds** (intensive care units, wards with high anti-infective prescription density) 2-4 x per week  Counselling by IDS via telephone  (unrestricted 24h/7d)  IDS consultation service on site (Mon. - Fri. 8am – 5pm) |
|  |  |

Differences between the study arms are shown in italics

**Supplementary Table 2a** ICATB.2 score (French)


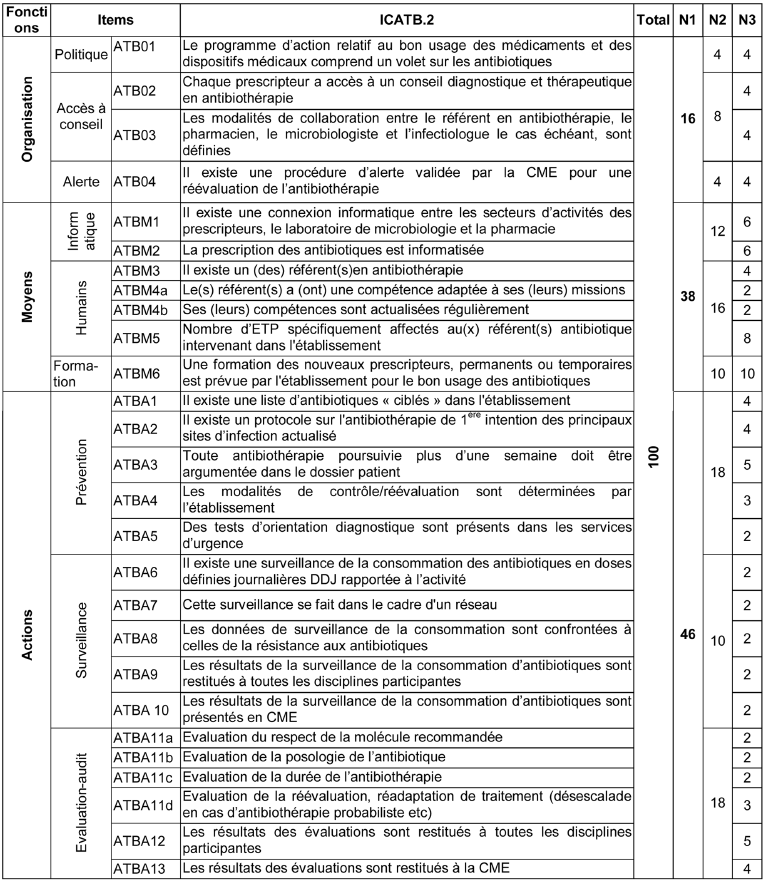


CME: Commission Médicale d'Établissement, ETP: equivalent temps plein

**Supplementary Table 2b** ICATB.2 score (English translation)

| **Functions** | **Items** | | **ICATB.2** | **Total** | **N1** | **N2** | **N3** |
| --- | --- | --- | --- | --- | --- | --- | --- |
| **Organisation** | Policy | ATB01 | The action programme on the proper use of medicines and medical devices includes a section on antibiotics | **100** | **16** | 4 | 4 |
|  | Access to counselling | ATB02 | All prescribers have access to diagnostic and therapeutic advice on antibiotic therapy |  |  | 8 | 4 |
|  |  | ATB03 | The terms of collaboration between AMS physician, pharmacist, microbiologist and ID specialist (if available) are defined. |  |  |  | 4 |
|  | Alert | ATB04 | There is an alert validated by the CME for the reassessment of antibiotic therapy. |  |  | 4 | 4 |
| **Resources** | IT | ATBM1 | There is an IT connection between the prescribers' sectors of activity, the microbiology laboratory and the pharmacy. |  | **38** | 12 | 6 |
|  |  | ATBM2 | Antibiotic prescriptions are computerised |  |  |  | 6 |
|  | Human | ATBM3 | There is one or more AMS physician |  |  | 16 | 4 |
|  |  | ATBM4a | The AMS physican(s) has (have) appropriate skills for their duties. |  |  |  | 2 |
|  |  | ATBM4b | The AMS physican(s) skills is (are) regularly updated |  |  |  | 2 |
|  |  | ATBM5 | FTEs specifically allocated to the AMS physican(s) |  |  |  | 8 |
|  | Training | ATBM6 | Training for new prescribers, whether permanent or temporary, is provided to ensure the proper use of antibiotics. |  |  | 10 | 10 |
| **Actions** | Prevention | ATBA1 | There is a list of "targeted" antibiotics in the establishment |  | **46** | 18 | 4 |
|  |  | ATBA2 | There is a guidance on 1st-line antibiotic therapy for the main sites of infection, which is updated regularly. |  |  |  | 4 |
|  |  | ATBA3 | Any antibiotic therapy continued for more than a week must be documented in the patient record. |  |  |  | 5 |
|  |  | ATBA4 | The monitoring/reassessment procedures are determined by the establishment. |  |  |  | 3 |
|  |  | ATBA5 | Diagnostic POC tests are available in the emergency department |  |  |  | 2 |
|  | Surveillance | ATBA6 | Antibiotic consumption is monitored in terms of defined daily doses (DDD) |  |  | 10 | 2 |
|  |  | ATBA7 | Antibiotic consumption monitoring is carried out as part of a network |  |  |  | 2 |
|  |  | ATBA8 | Antibiotic consumption monitoring data is compared with antibiotic resistance data |  |  |  | 2 |
|  |  | ATBA9 | Antibiotic consumption monitoring data are made available to all participating disciplines. |  |  |  | 2 |
|  |  | ATBA10 | Antibiotic consumption monitoring data are presented to the CME. |  |  |  | 2 |
|  | Evaluation-audit | ATBA11a | Evaluation of compliance with the recommended antibiotic |  |  | 18 | 2 |
|  |  | ATBA11b | Evaluation of antibiotic dosage |  |  |  | 2 |
|  |  | ATBA11c | Evaluation of the duration of antibiotic therapy |  |  |  | 2 |
|  |  | ATBA11d | Evaluation of reevaluation and adjustment of treatment (possible de-escalation etc.) |  |  |  | 3 |
|  |  | ATBA12 | The results of the evaluations are returned to all the participating disciplines. |  |  |  | 5 |
|  |  | ATBA13 | The results of the evluationss are presented to the CME |  |  |  | 4 |

CME: Commission Médicale d'Établissement = The CME is a committee of elected physicians in French hospitals, responsible for medical governance, FTE: full-time equivalent, POC: point of care

**
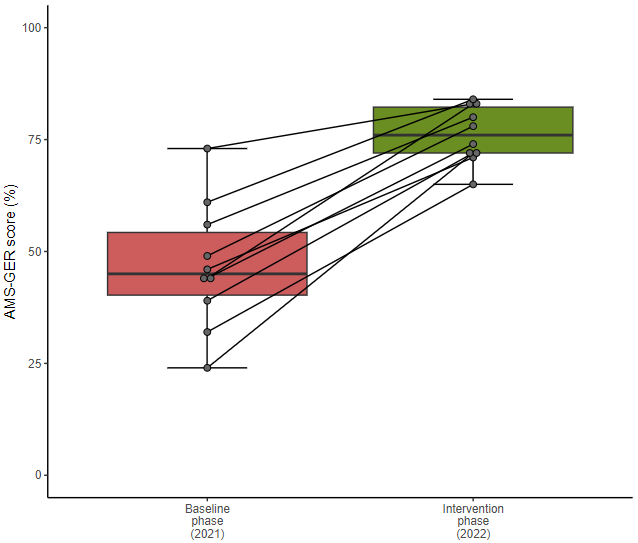
**

**Supplementary Fig. 1** AMS-GER score baseline vs. intervention phase with exclusion of PPS related questions

**
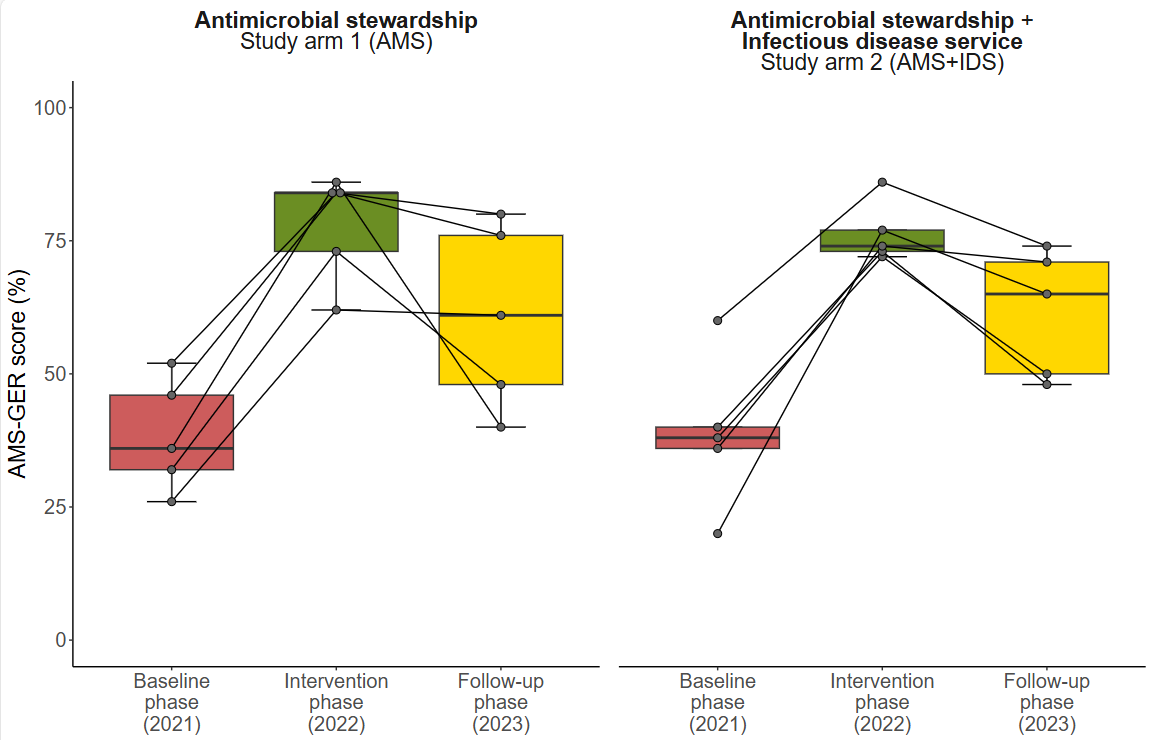
**

**Supplementary Fig. 2** AMS-GER score study arm 1 (AMS) vs. study arm 2 (AMS+IDS) baseline vs. intervention vs. follow-up phase


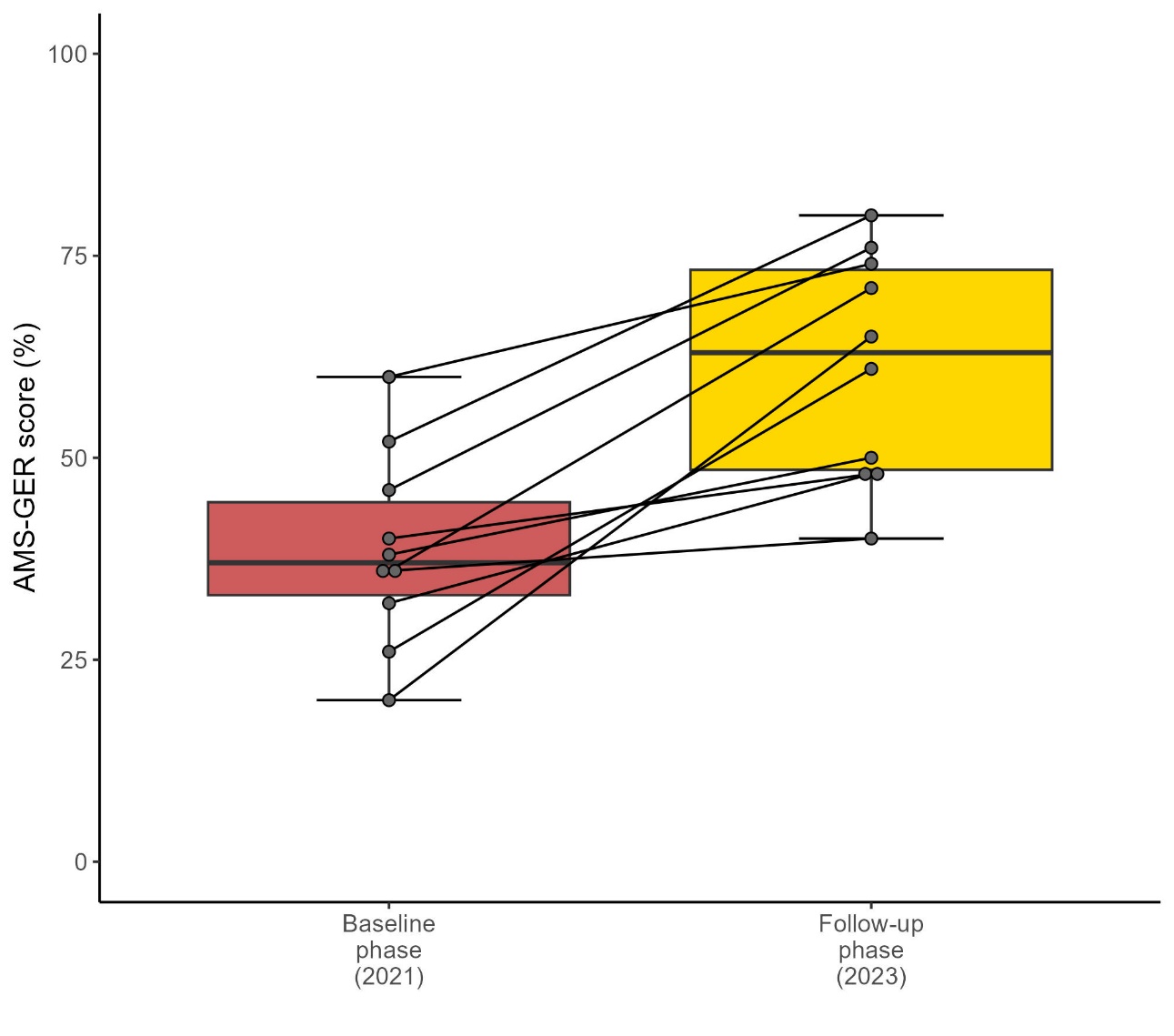


**Supplementary Fig. 3** AMS-GER score baseline vs. follow-up phase

A: Total AMS-GER score baseline vs. follow-up phase, B: Subcategory: framework, C: Subcategory: resources, D: Subcategory: action (prevention), E: Subcategory: action (surveillance) F: Subcategory: action (evaluation)

**
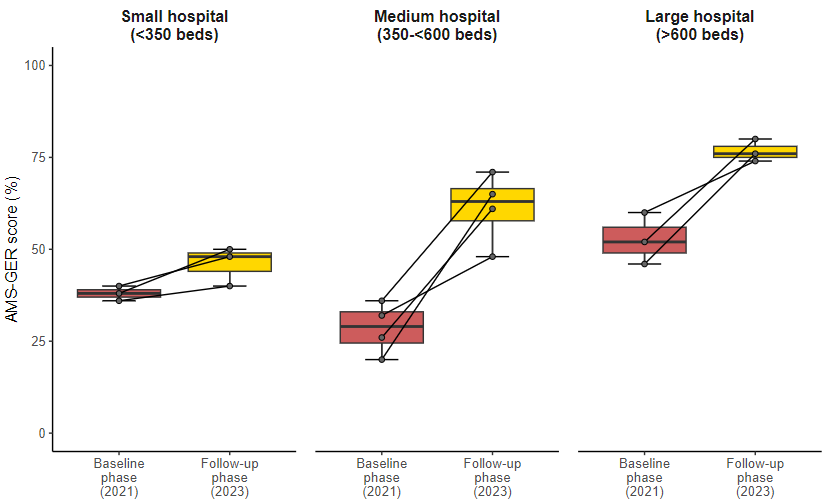
**

**Supplementary Fig. 4:** AMS-GER score baseline vs. follow-up phase regarding to hospital size

Small hospitals: <350 beds, n=3, medium hospitals: 350-600 beds, n=4, large hospitals >600 beds, n=3
